# Normalization of Patient-Identified Plasma Biomarkers in SMNΔ7 Mice following Postnatal SMN Restoration

**DOI:** 10.1371/journal.pone.0167077

**Published:** 2016-12-01

**Authors:** W. David Arnold, Sandra Duque, Chitra C. Iyer, Phillip Zaworski, Vicki L. McGovern, Shannon J. Taylor, Katharine M. von Herrmann, Dione T. Kobayashi, Karen S. Chen, Stephen J. Kolb, Sergey V. Paushkin, Arthur H. M. Burghes

**Affiliations:** 1 Department of Neurology, The Ohio State University Wexner Medical Center, Columbus Ohio, United States of America; 2 Department of Physical Medicine and Rehabilitation, The Ohio State University Wexner Medical Center, Columbus Ohio, United States of America; 3 VIB Center for the Biology of Disease – KU Leuven Department of Human Genetics, Leuven Belgium, United States of America; 4 Department of Biological Chemistry and Pharmacology, The Ohio State University Wexner Medical Center, Columbus Ohio, United States of America; 5 PharmOptima, Portage, Michigan, United States of America; 6 SMA Foundation, New York, New York, United States of America; Universita degli Studi di Torino, ITALY

## Abstract

**Introduction and Objective:**

Spinal muscular atrophy (SMA) is an autosomal recessive motor neuron disorder. SMA is caused by homozygous loss of the *SMN1* gene and retention of the *SMN2* gene resulting in reduced levels of full length SMN protein that are insufficient for motor neuron function. Various treatments that restore levels of SMN are currently in clinical trials and biomarkers are needed to determine the response to treatment. Here, we sought to investigate in SMA mice a set of plasma analytes, previously identified in patients with SMA to correlate with motor function. The goal was to determine whether levels of plasma markers were altered in the SMNΔ7 mouse model of SMA and whether postnatal SMN restoration resulted in normalization of the biomarkers.

**Methods:**

SMNΔ7 and control mice were treated with antisense oligonucleotides (ASO) targeting ISS-N1 to increase SMN protein from *SMN2* or scramble ASO (sham treatment) via intracerebroventricular injection on postnatal day 1 (P1). Brain, spinal cord, quadriceps muscle, and liver were analyzed for SMN protein levels at P12 and P90. Ten plasma biomarkers (a subset of biomarkers in the SMA-MAP panel available for analysis in mice) were analyzed in plasma obtained at P12, P30, and P90.

**Results:**

Of the eight plasma biomarkers assessed, 5 were significantly changed in sham treated SMNΔ7 mice compared to control mice and were normalized in SMNΔ7 mice treated with ASO.

**Conclusion:**

This study defines a subset of the SMA-MAP plasma biomarker panel that is abnormal in the most commonly used mouse model of SMA. Furthermore, some of these markers are responsive to postnatal SMN restoration. These findings support continued clinical development of these potential prognostic and pharmacodynamic biomarkers.

## Introduction

Spinal muscular atrophy (SMA) is an autosomal recessive disorder that results in the destruction of lower motor neurons and is the most common inherited cause of infant death [1, 2]. SMA is caused by low levels of SMN protein which is the result of homozygous loss of the *SMN1* gene and retention of a second closely related gene, *SMN2* [3–5]. The *SMN1* and *SMN2* genes differ by a single nucleotide in exon 7 a C to T change which results in alteration of a splice modulator resulting in the exclusion of SMN exon 7 from the majority of the transcript produced by *SMN2* [6–10]. SMN protein that lacks the amino acids encoded by exon 7 does not oligomerize well and gets rapidly degraded[11–13]. SMA has various severities (type 0–4) with type 0 having onset at birth, type 1 before the age of 6 months, type 2 before the age of 2 years and never gaining the ability to walk, type 3 patients gain the ability to walk and type 4 have an adult onset [14, 15]. Copy number of *SMN2* correlates with phenotypic severity, but there are exceptions to the copy number correlation [16–21]. One reason this can occur is due to the c.859G>C variant in exon 7 of *SMN2* which results in increased incorporation of *SMN2* exon 7 and thus increased amount of full length SMN mRNA produced [22–24]. This variant has been shown to not occur in type 1 cases, to be present in the heterozygote state with one additional *SMN2* copy (2 copy individual) in type 2 cases, and when this variant is present in 2 copies the individual has mild Type 3b SMA. This would indicate that approximately a 25% increase in full length SMN from a 2 copy *SMN2* SMA individual if given at the required time will result in a normal motor neuron function [25, 26].

Mouse models of SMA have been developed by placing the *SMN2* gene into the mice that have disruption of the mouse *Smn* gene [27–29]. SMA mouse models have been extensively used for testing SMA therapeutic strategies. Strategies to increase SMN protein have been developed that include small molecules that increase the incorporation of SMN exon7 [30, 31], antisense oligonucleotides that block negative regulators of SMN exon7 incorporation [32–34], and gene transfer of constructs producing full length SMN [35–38]. These different strategies have all shown major impact in mouse models of SMA [26, 39], and, in the case of gene therapy, also in a pig model of SMA [40]. Currently these potential treatments are being tested in several clinical trials [41] On the basis of preclinical results, there is very good evidence that these treatments will be effective provided that SMN levels are restored at the appropriate time in the required tissues.

To effectively implement SMN-related therapies in clinical trials, effective biomarkers are needed. Biomarkers can yield information regarding disease severity or prognosis (prognostic biomarkers), stratify patients regarding response to a particular therapy (predictive), and measure target engagement or therapeutic response with an intervention (pharmacodynamic). Putative candidates for biomarker application in patients with SMA have included an array of measures including electrophysiological measures, molecular markers, imaging studies, and other measures [42–46]. Clinical studies investigating electrophysiological measures such as compound muscle action potential (CMAP) and motor unit number estimation (MUNE) have shown good promise for these measures to determine disease severity and prognosis [43, 44, 47–50]. Additionally, preclinical studies have shown responsiveness of these markers with SMN restoration [40, 51–53]. A panel of protein markers has been identified that correlates with motor function scores in patients and thus tracks with the severity of the patient at a particular time point [54, 55]. The Biomarkers for SMA (BforSMA) study was a cross-sectional, single visit, multi-center, exploratory investigation that identified 200 candidate serum biomarkers [54]. From this, a 27 protein analyte biomarker panel (SMA-MAP) was subsequently validated [55]. However, whether these markers are indicative of disease severity and prognosis and also respond to treatment is unknown and untested.

In this study, we aimed to investigate 10 putative protein biomarkers in the SMNΔ7 mouse model of SMA (Jackson Lab catalogue number 5025) [29]. The panel of proteins was selected from proteins that were previously identified in cohorts of SMA patient to correlate with motor function [54, 55]. The goal of the study was to assess whether levels of these proteins are altered in SMNΔ7 mice. Furthermore, we inquired whether restoration of SMN levels postnatally would lead to normalization of these protein biomarkers. The ultimate goal of this work is to define whether these SMA biomarker proteins that correlate with disease severity could serve as prognostic biomarkers and also whether they respond to SMN restoration, and thus have potential as pharmacodynamic markers.

## Materials and Methods

### Ethics statement and experimental animals

This study was carried out in strict accordance with the recommendations in the Guide for the Care and Use of Laboratory Animals of the University Laboratory Animal Resources at The Ohio State University. Our protocol was approved by The Ohio State University Institutional Animal Care and Use Committee (IACUC), Office of Responsible Research Practices, under Permit Number 2008A0089. SMNΔ7 SMA mice (*SMN2*^+/+^; SMNΔ7^+/+^; *Smn*^−/−^) were generated by crossing phenotypically normal heterozygote mice (*SMN2*+/+; *SMNΔ7*+/+; *Smn*+/−) [29]. Heterozygote (*SMN2*+/+; *SMNΔ7*+/+; *Smn*+/−) (Het) mice were used as control animals. Neonatal mice were tattooed and tail snips were obtained for genotyping as previously described [34]. In all cases, the people collecting the data where blinded to genotype of mice and injection status was randomly assigned by a single person that was not responsible for data collection and analysis.

Anesthesia was administered with Isoflurane according to our animal protocol. Mice were humanely euthanized when exclusion criteria was met which included the inability of neonatal SMA mice to go to the mother to suckle (homing) and loss of greater than 20% of maximum weight that the particular animal achieved according to our IACUC approved animal protocol. Carbon Dioxide followed by cervical dislocation for secondary means of confirmation was used for euthanasia according to our approved protocol. When needed, steps were taken to minimize suffering with administration of systemic analgesia (ibuprofen) in the water bottle at 100mg/5ml providing a dose of approximately 30 mg/kg when needed.

### ASO intracerebroventricular injection

Antisense oligonucleotides (ASO) were delivered by intracerebroventricular injection as described previously[34]. Cohorts of SMA and Het mice were treated on P1 with 40 μg of morpholino ASO directed against ISS-N1 to increase full length SMN protein production from *SMN2* or scramble ASO on postnatal day 1 (P1) previously described by Porensky et al [34]. ASO directed against ISS-N1 increases median lifespan of the SMNΔ7 mouse from approximately 2 weeks to over 100 days with a single injection [34]. Treatment-genotypic groups of mice included SMA mice treated with ASO against ISS-N1 (ASO-SMA), SMA mice treated with scramble ASO (SMA), heterozygote mice treated with ASO against ISS-N1 (ASO-Het), heterozygote mice treated with scramble ASO (Het). This study included two cohorts of mice to allow endpoint whole blood, plasma, and tissue comparison at P12 in one cohort and longitudinal assessment out to 90 days in another cohort. Untreated SMA mice were only available for comparison at P12 due to a median survival of about 2 weeks [29]. All injections were performed by VLM.

### Whole blood and tissue sampling

The P12 takedown cohort included endpoint tissue and whole blood sample collection at P12 from Scramble-treated Het, ASO-treated Het, ASO-treated SMA, and scramble-treated SMA. In this P12 cohort, the levels of 10 putative SMA biomarkers in plasma and SMN levels in mouse tissue homogenates and whole blood samples from P12 Het and SMA mice in response to drug treatment were assessed. This allowed comparison to untreated SMA mice. The longitudinal cohort included ASO-treated SMA and ASO-treated and Scramble-treated control mice underwent an orbital bleeds for a volume of 100–150μL at P30 and P90 followed by endpoint tissue harvesting at P90 of the quadriceps muscle, liver, spinal cord, and brain. All samples where encoded and the analysis of protein levels was performed blinded to genotype and treatment.

### Electrophysiological recordings

Electrophysiological recordings including both compound muscle action potential (CMAP) and motor unit number estimation (MUNE) were obtained from the right sciatic-innervated triceps surae muscle as previously described [52, 56, 57]. CMAP amplitude and MUNE were compared between ASO-treated SMA mice and ASO-treated controls at P12, P30, and P90. WDA performed the MUNE and CMAP and was blinded to genotype of the mice.

### SMN ECL immunoassay

SMN levels were evaluated in brain, liver, spinal cord and quadriceps muscle at P12 and P90. Tissue samples were received frozen and were maintained at -80°C until homogenization with a VWR Powermax AHS200 homogenizer. ER4 buffer (50 mM Tris, pH 7.5, 300 mM NaCl, 10% glycerol, 3 mM EDTA, 1 mM MgCl_2_, 1% Triton X-100, 20 mM β-glycerophosphate, 25 mM NaF) containing protease inhibitors (0.5 μl cocktail/ml ER4 buffer) was added to pre-weighed tissue samples using the respective ER4 buffer/tissue ratio shown in Table 1. Homogenates were clarified via centrifugation at 20,000 x g for 10 minutes at 4°C. Clarified supernatants were frozen and maintained at -80°C until the time of assay. SMN ECL Immunoassays were carried out using methods developed by PharmOptima.

**Table 1:** Plasma dilutions.

Biomarker protein	Assay Range (pg/mL)	Plasma dilution
Fetuin A	61–250,000	1:640
Tetranectin	0.24–1000	1:160
IGF-1	2.44–10,000	1:160
Cadherin	97.6–400,000	1:320
Vitronectin	24–100,000	1:640
CHI3L1	2.44–10,000	1:160
DPPIV	2.44–10,000	1:160
COMP	2.44–10,000	1:160
AXL	2.44–10,000	1:160
SPP1	2.44–10,000	1:640

Prior to the assay, tissue homogenates were thawed to room temperature and the resultant lysate was diluted in sample dilution buffer. Final dilutions are shown in Table 2. Plates were read using an MSD 6000 Sector Imager (Meso Scale Discovery). Data reduction from the SMN ECL Immunoassay was performed using software provided with the MSD 6000 Imager. Resultant SMN values were normalized to total soluble protein and reported as pg SMN per mg soluble protein. Total soluble protein present in clarified supernatants was determined using the BCA protein assay kit (Pierce) according to the manufacturer’s instructions. Extracts were diluted into distilled water using the dilutions described for the respective tissues in Table 2.

**Table 2:** Tissue homogenization in ER4 lysis buffer and dilutions for protein and ELISA assays.

Tissue	ER4:tissue homogenization ratio (μl/mg tissue)	Powermax cycles	Protein assay dilution in water	ELISA assay dilution in assay buffer
Brain	10	2 X 5 sec	1:40	1:320
Liver	10	2 X 5 sec	1:40	1:320
Quadriceps	10	2 X 10 sec	1:25	1:40
Spinal cord	10	2 X 5 sec	1:20	1:80

SMN levels were evaluated in whole blood at P12, P30, and P90. Whole blood samples were received frozen and were maintained at -80°C until assay. SMN ECL Immunoassay was carried out using methods developed for whole blood by PharmOptima and described in Zaworski *et al* 2016[58]. Prior to the assay, whole blood was thawed to room temperature and the resultant lysate was diluted 1:160 using assay sample dilution buffer. SMN values were reported as pg SMN per ml whole blood.

### Plasma biomarker proteins

The protein analytes assayed included the following proteins: fetuin A, osteopontin (SPP1), vitronectin, AXL kinase (AXL), chitinase-3-like-1 (CHI3L1), cartilage oligomeric matrix protein (COMP), dipeptidyl-dipeptidase 4 (DPPIV), tetranectin, insulin-like growth factor 1 (IGF-1), and cadherin 13. All samples for each biomarker were assayed in duplicate and the average used for analysis. The mouse 10-plex assay panel was developed for Spinal Muscular Atrophy Foundation by PharmOptima using commercially available antibody and calibrator reagents (S1 Table) and multiplex assay plates used in this study were custom manufactured by Meso Scale Discovery.

Assays used included the following: 3 plex assay: Fetuin A, Osteopontin (SPP1) and Vitronectin; 5 plex assay: AXL kinase, Chitinase-3-like-1 (CHI3L1), Cartilage oligomeric matrix protein (COMP), Dipeptidyl-dipeptidase 4 (DPPIV) and Tetranectin; Singleplex Assays: Insulin like growth factor 1 (IGF-1) and Cadherin 13. The protein standards used for COMP and tetranectin are recombinant human proteins. For this reason the protein concentrations obtained for COMP and tetranectin were interpreted as relative rather than absolute quantities.

### MSD assay protocol

All calibrators (blended and in singlet), detection antibodies (blended and in singlet), plasma and serum samples, and secondary (detection) antibody and streptavidin preparations were prepared in 1% bovine serum albumin (BSA), 0.05% Tween 20, 1X PBS, pH 7.4 (Blocker A) as the diluent. SULFO-TAG streptavidin (500 μg/ml) was diluted 1:1000 in 1% Blocker A prior to use. Sample and detection antibody volumes were 25 μl per well. Plates were washed 3 times with 200 μl Tris buffered Saline, 0.05% Tween (TTBS), pH 7.5 between incubation steps. Following incubation steps 150 μl of 1X Read Buffer T was added to all wells and plates were read using an MSD Sector^®^ Imager 6000. Biomarker levels were calculated directly from the standard curves using MSD software provided with the MSD Imager

Plasma samples were diluted into 1% Blocker A to the final dilutions shown in Table 1. For the 1:160 plasma dilution 5 μl of plasma was diluted into 795 μl of 1% Blocker A. For the 1:320 plasma dilution 2 μl of plasma was diluted into 638 μl of 1% Blocker A. For the 1:640 plasma dilution 100 μl of the 1:160 plasma dilution was diluted into 300 μl of 1% Blocker A. Twenty-five microliters of the respective dilutions were used per well of the assay plate.

### IGF-1 plasma sample preparation

Most of the IGF-1 in serum or plasma is complexed with binding proteins which may mask the protein from capture and detection antibodies. In order to disrupt this interaction plasma samples were extracted using low pH and ethanol as a solvent. The procedure used is described in Enzolife sciences IGF-1 ELISA assay kit manual (cat # ADI-900-150). Final dilution for IGF-1 prior to assay was 1:160 in 1% Blocker A.

### Plasma extraction procedure for IGF-1 samples

10 μl of plasma was diluted to 50 μl with 0.25 N HCl in 87.5% ethanol. The samples were mixed, incubated at room temperature for 30 minutes and spun down at 10,000g for 10 minutes. 50 μl of supernatant was added to 50 μl of neutralization solution (1M Tris-HCl, pH 8). 24 μl of resultant neutralized sample was then added to 296 μl of 1% Blocker A solution (1% bovine serum albumin (BSA), 0.05% Tween 20, 1X PBS, pH 7.4). In total the sample was diluted 1:160.

### Statistical analysis

Comparisons between groups were performed using One-way ANOVA and Dunnett's multiple comparisons test (GraphPad Prism, La Jolla CA). Tukey post-hoc tests were utilized to determine significance among the groups. For one-way ANOVA comparison of plasma biomarker levels, ASO-SMA, SMA, and Het mice were compared against ASO-Het mice. For one-way ANOVA comparison of SMN levels in tissues and blood SMA, ASO-Het, and Het mice were compared against ASO-SMA mice. For correlation analysis, standard Pearson test was performed including data for all mice and treatments (SMA, ASO-SMA, ASO-Het, and Het). For all studies, p<0.05 was considered significant. All values were shown as mean±standard deviation.

## Results

### SMN levels in tissues at P12 and P90 and whole blood at P12, P30, and P90

**Fig 1:**
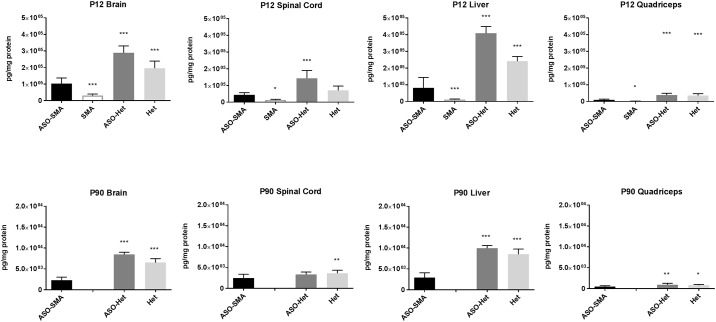
SMN levels in brain, spinal cord, liver and quadriceps muscle at P12 and P90. SMN levels were compared between ASO-treated SMA mice and untreated SMA, ASO-treated Het mice, and untreated Het mice. At P12, SMN levels were increased in ASO-SMA mice (n = 12) in all tissues compared with SMA mice (n = 13). SMN levels were decreased in ASO-SMA mice compared with ASO-Het (n = 10) and Het (n = 5) for all tissues except for spinal cord for which there was not significant difference between ASO-SMA and Het. At P90, SMN levels in ASO-SMA (n = 12) were diminished in all tissues compared with ASO-Het (n = 7) and Het (n = 8) animals except spinal cord for which there was no significant difference between ASO-SMA and controls. (Note the different scale for P12 versus P90 SMN levels).

SMN levels were increased in all tissues at P12, including peripheral tissues such as liver and muscles, in ASO-SMA in response to P0 ICV administration of ISS-N1 when compared to SMA mice (Fig 1). Yet, SMN levels in ASO-SMA mice at P12 were significantly lower than both ASO-Het and Het animals. At P90 untreated SMA mice are not available for comparison (mean survival ~2 weeks) [29]. SMN levels in ASO-SMA at P90 were reduced compared with treated (ASO-Het) and untreated (Het) animals for all tissues except spinal cord (Fig 1). Interestingly, there was dramatic variability between the various tissues as well as a reduction of SMN expression (in all tissues) in mice at P90 compared with mice at P12.

The average levels of SMN in the various tissues at P12 were compared in SMA and ASO-mice mice to determine magnitude of change with ASO treatment. We also examined levels of SMN compared between ASO-SMA and untreated Het to understand how “normalized” levels of SMN were in ASO-treated mice. SMN level increase in ASO-SMA mice was 3.8 fold in the brain, 2.9 fold in the spinal cord, 3.0 fold in the blood, and 6.5 fold in the liver compared with SMA mice. Furthermore, to understand how these levels compared in the untreated Het versus ASO-SMA mice SMN level ratios were calculated (ASO-SMA/Untreated Het) using the mean values for each group. The SMN level ratios were 0.6 for the brain and spinal cord, 0.4 for the liver, and 0.2 for whole blood. Therefore, as expected, while the fold change for SMN levels was the greatest in liver, the levels relative to untreated Het animals were more similar to het mice in the brain and spinal as compared to the liver and whole blood.

**Fig 2:**
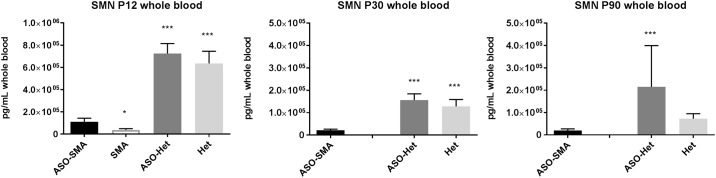
Whole blood SMN levels at P12, P30, and P90. SMN levels were between ASO-treated SMA mice and untreated SMA, ASO-treated Het mice, and untreated Het mice. At P12, SMN levels were increased in ASO-SMA mice (n = 11) compared to SMA mice (n = 13) but reduced compared to ASO-Het (n = 10) and Het mice (n = 4). Untreated SMA mice have a mean survival of ~2 weeks. Therefore no SMA disease control animals are available for comparison at P30 or P90. At P30 SMN levels were diminished in ASO-SMA mice (n = 10) compared to ASO-Het (n = 5) and Het (n = 8) mice. At P90, SMN levels in ASO-SMA mice (n = 11) were reduced compared with ASO-Het (n = 6) but not Het (n = 8) mice. *, p<0.05, ***, p<0.001 (Note the different scale for P12, P30, and P90 SMN levels).

Whole blood samples at P12 showed increased SMN levels in treated SMA mice (ASO-SMA) compared to scramble treated SMA mice (SMA) (Fig 2). Similar to tissue levels of SMN, whole blood levels of SMN were significantly lower in ASO-SMA mice compared with both ASO-Het and Het animals at P12. At P30 whole blood levels of SMN are reduced in ASO-SMA compared to both ASO-Het and Het mice. At P90, whole blood SMN levels were significantly lower in ASO-SMA compared to ASO-Het animals, however scramble treated Het animals did not differ from ASO-Het animals.

### Response of plasma protein analyte panel to SMA phenotype and postnatal SMN restoration

**Fig 3:**
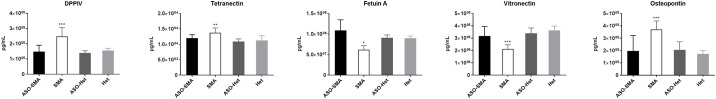
SMN-Responsive analytes. Levels of biomarker analytes were compared at P12 in ASO-SMA mice (n = 12), in SMA mice (n = 13), ASO-Het (n = 10), and Het mice (n = 5). ASO-Het mice were considered controls for statistical comparison using One-way ANOVA and Dunnett's multiple comparisons test (GraphPad Prism, La Jolla CA). Statistical differences between cohorts and controls are shown as * <0.05, ** <0.01, *** <0.001.

Five of the 10 protein analytes were responsive to SMN restoration in SMA but not in heterozygous mice including DPPIV, tetranectin, fetuin A, osteopontin, and vitronectin. These analytes showed both significant differences in SMA mice compared to controls and normalization in ASO-treated SMA mice (Fig 3).

**Fig 4:**
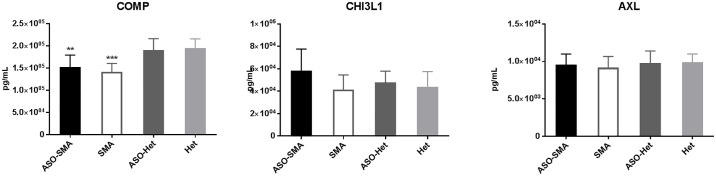
Analytes that are not responsive to SMN. ASO-Het mice were considered controls for statistical comparison using One-way ANOVA and Dunnett's multiple comparisons test (GraphPad Prism, La Jolla CA). COMP was significantly reduced in ASO-SMA (n = 12) and SMA (n = 13) compared with ASO-Het (n = 10). There was no significant difference between Het (n = 5) and ASO-Het mice for COMP. AXL and CHI3LI were unchanged in all groups compared with ASO-Het mice. ** <0.01, *** <0.001.

Of the remaining protein analytes, AXL and CHI3LI were not changed in SMA and COMP was altered in SMA mice but ASO-treated SMA mice were similarly altered (i.e. nonresponsive to SMN) (Fig 4). There was insufficient sample to allow adequate comparison across all groups for Cadherin and IGF-1, but IGF-1 showed significant reduced in ASO-SMA mice compared to controls (ASO-Het) (S1 Fig).

### Correlation of plasma protein analytes and smn levels

The plasma protein analytes were analyzed for correlation with SMN levels in brain, spinal cord, liver and quadriceps muscle at P12 and P90. Four of the 5 SMN-responsive analytes showed good correlation with SMN levels in brain, spinal cord, liver, and quadriceps muscle at P12 (S2 Table). Whereas, fetuin A levels did not show significant correlation with SMN levels in any tissues at P12. At P90, osteopontin showed good correlation with spinal cord SMN levels, and tetranectin was correlated with spinal cord, liver and quadriceps SMN levels (S3 Table).

Plasma protein analytes were also analyzed for correlation with whole blood SMN levels at P12, P30, and P90 (S4 Table). Osteopontin and vitronectin showed good correlation with whole blood SMN levels at P12, P30, and P90. DPPIV and tetranectin showed good correlation at only P12 but not P30 or P90. Similar to SMN levels in tissues, fetuin A did not show correlation with SMN levels in whole blood at P12, P30, and P90.

### Electrophysiological measures and correlation with plasma protein analytes

**Fig 5:**
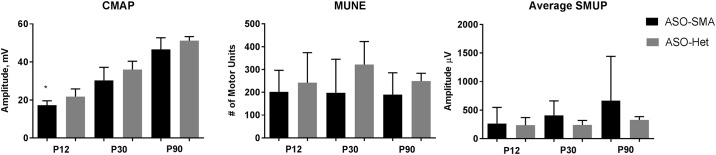
Longitudinal electrophysiology. At P12 CMAP amplitude is reduced in ASO-SMA mice (n = 5) compared with ASO-Het (n = 8) (p = 0.045). Otherwise there are no statistically significant differences. Compound muscle action potential, CMAP; motor unit number estimation, MUNE; and average Single Motor Unit Potential, SMUP.

We previously published the effects of SMN restoration on CMAP and MUNE responses showing restoration of MUNE at P12 and both CMAP and MUNE by P30 [59]. Blood samples were obtained from some of the animals from this previously published study for analysis of plasma analytes. Consistent with our prior study, CMAP, MUNE, and SMUP comparison between ASO-SMA and ASO-Het showed statistically significant difference between groups for CMAP at the P12 measurement, but no other parameter showed a significant change between groups (Fig 5) [52]. Plasma protein analytes were correlated with electrophysiological measures at P30 and P90. No whole blood was available at P12 in this cohort of animals as the mice were studied longitudinally. At P30, vitronectin showed moderate negative correlation with P30 CMAP (r = -0.622; p = 0.031). SMUP and MUNE showed no significant correlation with any of the plasma protein analytes at P30. There were no significant correlations between the plasma protein analytes and electrophysiological measures at P90.

### Longitudinal measures

**Fig 6:**
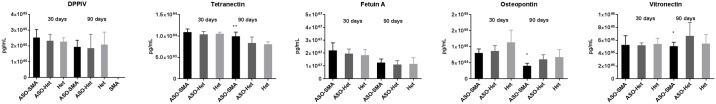
Longitudinal Measures of SMN-corrected biomarkers. DPPIV and fetuin A showed no statistically significant change compared to ASO-Het and Het mice at either P30 or P90. The other 3 analytes, tetranectin, osteopontin, and vitronectin, showed no change at P30 but were all altered at P90. * <0.05, ** <0.01.

The 5 plasma protein biomarker analytes that were responsive to both SMA phenotype and SMN restoration were analyzed longitudinally to assess stability over time (Fig 6). Two of the 5 plasma protein analytes (DPPIV and Fetuin A) showed no statistically significant change compared to ASO-Het and Het mice at either P30 or P90. The other 3 protein analytes (tetranectin, osteopontin, and vitronectin) showed no change at P30 but were altered at P90. It is expected that plasma analytes most tightly associated with effects of changing SMN levels would be altered at the P90 time point as morpholino ASO efficacy is diminished 60 days post-injection per our prior work and as shown in Figs 1 and 2 [34]. Interestingly, at the P90 time point, tetranectin and vitronectin were changed in the same direction in ASO-SMA compared to ASO-Het and Het mice as noted in untreated SMA at P12 (compared to ASO-SMA, ASO-Het, and Het) when the efficacy of the morpholino ASO would be expected to be diminished. In contrast, osteopontin was increased in SMA mice at P12 compared ASO-Het mice, but at P90 osteopontin was decreased in ASO-SMA mice compared with ASO-Het.

## Discussion

Meaningful translational biomarkers in SMA would predict future clinical severity and provide readout of response to therapy. A suitable molecular biomarker would correlate with function in humans with SMA and are altered in mouse models of SMA. A potential pharmacodynamic biomarker would respond to SMN restoration that results in amelioration of the SMA phenotype and functional improvements. Here, we were able to assess 8 protein analytes from the SMA-MAP panel in the SMNΔ7 mouse model of SMA. Our primary goal was to test the pharmacodynamic responsiveness of these markers to an experimental SMA therapy in particular when given early when there is a known positive response of these mice to treatment. First, we showed that levels are altered in SMA mice for 6 of the 8 analytes tested. Furthermore, we show that of these, 5 molecular markers are responsive to SMN restoration and correction of SMA phenotype.

We recently reported that a subset of the SMA-MAP analyte panel were altered in infants with SMA aged 6 months or less compared with age matched healthy infants [43]. When we compare the results of our mouse study with the results of the SMA infant study, 2 of the 5 SMN-responsive protein analytes in the mouse study, DPPIV and tetranectin, showed the same direction of change in SMA mice compared with the infants with SMA, 2 were not changed in SMA infants, and 1 was not performed (Table 3). Interestingly, osteopontin, DPPIV, and tetranectin, which were increased in SMA mice, were previously shown in to have a positive correlation with the MHMFS in patients with SMA (i.e. the levels of protein were increased in association with increased function) [54, 55]. Similarly, fetuin A and vitronectin which were reduced in SMA mice in our study were previously shown to have a negative correlation with function in SMA patients (i.e. the levels of protein were reduced in association with increased function) [54, 55].

**Table 3:** Biomarkers in SMA Mice, SMN-restored Mice, and Human SMA.

Biomarker	Change in SMA mice	Normalized with SMN	Correlation with MHFMS(Kobayashi et al. 2013)	Change in SMA (<6 months) (Kolb et al. 2016)
Osteopontin	Increased	Yes	Direct	No change
DPPIV	Increased	Yes	Direct	Increased
Tetranectin	Increased	Yes	Direct	Increased
Fetuin A	Decreased	Yes	Inverse	No change
Vitronectin	Decreased	Yes	Inverse	Not performed

Overall, the changes in these protein analytes (in both mice and human studies) together with the pattern of correlation (i.e. direct or indirect) with the MHMFS suggest that the changes in the biomarkers represent compensatory changes rather than changes directly attributable to SMA. For instance, tetranectin is increased in SMA mice and infants with SMA. Yet, increased levels of tetranectin correlate with improved performance on the MHMFS [54, 55]. Therefore, tetranectin appears to increase in compensation to the SMA phenotype. Tetranectin has also shown utility as a biomarker in other disorders such as coronary heart disease, and a decrease in tetranectin is associated with increased numbers of affected vessels [60]. DPPIV was also shown to be abnormal in SMA and normalized in ASO-SMA mice. DPPIV is known to play a role in glucose metabolism being responsible for the degradation of incretins [61]. Glucose metabolism has been reported to be disrupted in SMA, but whether it is directly related to SMN deficiency or a secondary consequence of muscle wasting is less clear [62]. The molecule, Fetuin A, has been used as a biomarker of multiple sclerosis using cerebrospinal fluid samples [63]. While the response of these markers to treatment does not mean the markers are directly related to the pathophysiological mechanism of SMA, they may be used to follow a response to treatment. Indeed, these plasma protein analytes may also have utility in other disorders affecting motor function.

In this study, we also followed the levels of plasma protein analytes longitudinally for change over time. At P30, the effects of morpholino ASO are persistent [34]. In contrast, at P90 the effects of morpholino ASO are reduced [34]. Interestingly, of the 5 responsive analytes, tetranectin, vitronectin, and osteopontin showed a later statistically significant change at P90. Of these three, only tetranectin and vitronectin showed a similar direction of change in the ASO-SMA mice at P90 as compared with untreated SMA mice at P12. Therefore, the response of osteopontin levels to SMN levels may be less specific or may be related to differential bone remodeling at various stages of development in neonatal and adult mice[64]. Altogether, the datasupport that tetranectin and vitronectin are potential pharmacodynamic biomarkers and could be used to follow response to treatment sufficiency over time (i.e. identify the need for retreatment). Furthermore, vitronectin showed correlation with a measure of motor function (CMAP amplitude) at P30 further suggesting that this analyte would be an appropriate biomarker.

The SMN restoration paradigm utilized in the current study was ASO delivered by ICV injection. In these studies, ASO is clearly distributed in a systemic distribution as highlighted by the increases in SMN in the various tissues in ASO-SMA mice compared to SMA mice at P12. Yet, the primary target in these mice was the central nervous system. Thus, systemic restoration could be expected to change biomarkers in varying magnitudes compared with the method of delivery presented here. This is important, as the precise tissue-specific sensitivity to low SMN and SMN restoration is undefined in human SMA. Interestingly in the current studies, SMN expression varied between tissues and at different ages in treated and untreated SMA and control mice. It will be important in the future to determine which of the SMA-MAP panel or combination of the markers from the panel respond in human patients with early and latter SMN restoration therapy and how this correlates with disease outcome. The results of our studies support the use of the SMA-MAP panel in future therapeutic SMA clinical trials.

## Supporting Information

S1 Fig Analytes with insufficient data for comparison between groups. ASO-Het mice were considered controls for statistical comparison using One-way ANOVA and Dunnett's multiple comparisons test (GraphPad Prism, La Jolla CA). ASO-SMA (n = 5) had significant lower IGF-1 levels compared with ASO-Het (n = 10). There was no significant difference between ASO-Het and Het animals (n = 11) IGF-1 was only assessed in one SMA mouse. Cadherin showed no significant difference between ASO-SMA (55894 ± 38120 pg/mL; n = 6) and SMA(56456 ± 52184 pg/mL; n = 9) (p = 0.51, unpaired t-test), but results in ASO-Het or Het mice were not available for comparison. *** <0.001.
(DOCX)

S1 Table Antibody and calibrator reagents. (DOCX)

S2 Table Correlations between responsive plasma analytes and SMN levels of various tissues in mice at P12. Shaded boxes represent significant values at p<0.05. ASO-SMA n = 12, SMA n = 13, ASO-Het n = 10, Het n = 5.
(DOCX)

S3 Table Correlations between responsive plasma analytes and SMN levels of various tissues in mice at P90. ASO-SMA n = 5, ASO-Het n = 7, Het n = 8. Shaded boxes represent significant values at p<0.05.
(DOCX)

S4 Table Correlation of whole blood SMN and plasma analyte levels. P12: ASO-SMA n = 12, scramble SMA n = 13, ASO-Het n = 10, scramble Het n = 5; P30/P90: ASO-SMA n = 12, ASO-Het n = 7, scramble Het n = 8. Shaded boxes represent significant values at p<0.05.
(DOCX)

S5 Table Raw data tables for plasma biomarkers, SMN Levels and Electrophysiology at P12, P30, and P90. Biomarker concentration is normalized to total soluble protein (pg/ml). Concentration of SMN protein in tissues is normalized to total soluble protein (pg/mg). Concentration of SMN in whole blood is pg SMN per ml whole blood. CMAP, compound muscle action potential, MUNE, motor unit number estimation, SMUP, single motor unit potential.
(DOCX)
