# Supplementary figures and images for: Normalization of Patient-Identified Plasma Biomarkers in SMNΔ7 Mice following Postnatal SMN Restoration
Source: PLoS One. 2016 Dec 1;11(12):e0167077. doi: 10.1371/journal.pone.0167077 (PMC5132001; doi:10.1371/journal.pone.0167077)

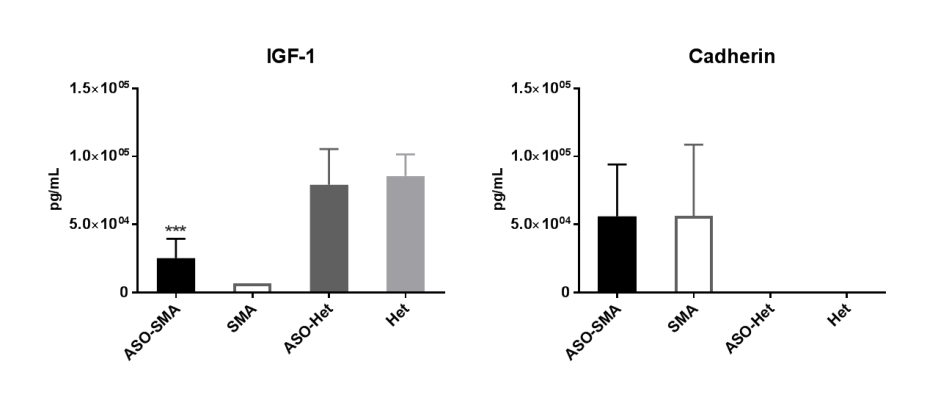

Supplement: S1 Fig — ASO-Het mice were considered controls for statistical comparison using One-way ANOVA and Dunnett's multiple comparisons test (GraphPad Prism, La Jolla CA). ASO-SMA (n = 5) had significant lower IGF-1 levels compared with ASO-Het (n = 10). There was no significant difference between ASO-Het and Het animals (n = 11) IGF-1 was only assessed in one SMA mouse. Cadherin showed no significant difference between ASO-SMA (55894 ± 38120 pg/mL; n = 6) and SMA(56456 ± 52184 pg/mL; n = 9) (p = 0.51, unpaired t-test), but results in ASO-Het or Het mice were not available for comparison. *** <0.001. (DOCX) [file pone.0167077.s001.docx]
