# Supplementary material for: Normalization of Patient-Identified Plasma Biomarkers in SMNΔ7 Mice following Postnatal SMN Restoration
Source: PLoS One. 2016 Dec 1;11(12):e0167077. doi: 10.1371/journal.pone.0167077 (PMC5132001; doi:10.1371/journal.pone.0167077)
Supplement: S1 Table — (DOCX) [file pone.0167077.s002.docx]

**S1 Table: Antibody and calibrator reagents**

| **Biomarker** | **Calibrator** | **1^o^ Capture Antibody** | **2^o^ Detection Antibody** |
| --- | --- | --- | --- |
| Vitronectin (VTN) | Cell Sciences CRV128B | Cell Sciences CSI20157A Rabbit polyclonal anti-mouse | Cell Sciences CSI20041A Biotinylated Rabbit polyclonal anti-mouse |
| Cartilage oligomeric matrix protein (COMP) | R&D Systems 3134-CP-050 | R&D Systems AF3134 Goat polyclonal anti-human | R&D Systems AF3134 Goat polyclonal anti-human |
| Cadherin (CDH13): | R&D Systems 6768-CA-050 | Goat polyclonal anti-human CDH13 R&D Systems AF3264 | Sulfo-tagged Goat polyclonal anti-human CDH13 R&D Systems AF3264 (sulfotagginig performed by Pharmoptima) |
| Tetranectin (CLEC3B): | R&D Systems 5170-CL-050 | R&D Systems AF5170 Sheep polyclonal anti-human tetranectin | R&D Systems BAF5170 Biotinylated Sheep polyclonal anti-human tetranectin |
| AXL | R&D Systems 854-AX-100 | R&D Systems MAB854 rat anti-mouse AXL | R&D Systems BAF854 biotinylated goat anti mouse AXL |
| CD26/DPPIV | R&D Systems 954-SE | R&D Systems MAB954 rat anti-mouse DPPIV | R&D Systems BAF954 biotinylated goat anti mouse DPPIV |
| Fetuin A (alpha-2HS-glycoprotein) | R&D Systems CF-1563-PI-050 | R&D Systems MAB15632 rat anti-mouse Fetuin A | R&D Systems BAM15631 biotinylated rat anti-mouse Fetuin A |
| insulin-like growth factor 1 (IGF-1) | R&D Systems 791-MG | R&D Systems MAB791 Hamster anti-mouse IGF-1 | R&S Systems BAF791 biotinylated goat anti-mouse IGF-1 |
| osteopontin (SPP1) | R&D Systems 2649-CH-050 | R&S Systems AF808 goat anti-mouse SPP1 | R&D Systems BAF808 biotinylated goat anti-mouse SPP1 |
| chitinase-3-like-protein 1 (CHI3L1) | R&D Systems 2649-CH-050 | R&D Systems AF2649 sheep anti-mouse CHI3L1 | R&D Systems BAF2649 biotinylated sheep anti-mouse CHI3L1 |
