# Supplementary material for: Normalization of Patient-Identified Plasma Biomarkers in SMNΔ7 Mice following Postnatal SMN Restoration
Source: PLoS One. 2016 Dec 1;11(12):e0167077. doi: 10.1371/journal.pone.0167077 (PMC5132001; doi:10.1371/journal.pone.0167077)
Supplement: S2 Table — Shaded boxes represent significant values at p<0.05. ASO-SMA n = 12, SMA n = 13, ASO-Het n = 10, Het n = 5. (DOCX) [file pone.0167077.s003.docx]

**S3 Table: Correlations between responsive plasma analytes and SMN levels of various tissues in mice at P12**

| **P12** | **Brain** | | **Spinal Cord** | | **Liver** | | **Quad** | |
| --- | --- | --- | --- | --- | --- | --- | --- | --- |
|  | **r** | **p-value** | **r** | **p-value** | **r** | **p-value** | **r** | **p-value** |
| Osteopontin | **-0.543** | **<0.001** | **-0.516** | **<0.001** | **-0.481** | **0.002** | **-0.499** | **0.001** |
| DPPIV | **-0.650** | **<0.001** | **-0.608** | **<0.001** | **-0.599** | **<0.001** | **-0.623** | **<0.001** |
| Tetranectin | **-0.667** | **<0.001** | **-0.555** | **<0.001** | **-0.634** | **<0.001** | **-0.657** | **<0.001** |
| Fetuin A | 0.285 | 0.0752 | 0.266 | 0.097 | 0.152 | 0.348 | 0.240 | 0.136 |
| Vitronectin | **0.603** | **<0.001** | **0.546** | **<0.001** | **0.505** | **<0.001** | **0.596** | **<0.001** |
