# Supplementary material for: Normalization of Patient-Identified Plasma Biomarkers in SMNΔ7 Mice following Postnatal SMN Restoration
Source: PLoS One. 2016 Dec 1;11(12):e0167077. doi: 10.1371/journal.pone.0167077 (PMC5132001; doi:10.1371/journal.pone.0167077)
Supplement: S3 Table — ASO-SMA n = 5, ASO-Het n = 7, Het n = 8. Shaded boxes represent significant values at p<0.05. (DOCX) [file pone.0167077.s004.docx]

**S4 Table: Correlations between responsive plasma analytes and SMN levels of various tissues in mice at P90**

| **P90** | **Brain** | | **Spinal Cord** | | **Liver** | | **Quad** | |
| --- | --- | --- | --- | --- | --- | --- | --- | --- |
|  | **r** | **p-value** | **r** | **p-value** | **r** | **p-value** | **r** | **p-value** |
| Osteopontin | 0.237 | 0.315 | **0.468** | **0.037** | 0.439 | 0.053 | 0.418 | 0.067 |
| DPPIV | 0.132 | 0.579 | -0.041 | 0.862 | 0.051 | 0.830 | -0.052 | 0.828 |
| Tetranectin | -0.422 | 0.064 | **-0.650** | **0.002** | **-0.535** | **0.017** | **-0.563** | **0.010** |
| Fetuin A | -0.197 | 0.405 | -0.299 | 0.201 | -0.337 | 0.146 | -0.29 | 0.157 |
| Vitronectin | 0.161 | 0.498 | 0.286 | 0.222 | 0.223 | 0.345 | 0.460 | 0.041 |
