# Supplementary material for: Normalization of Patient-Identified Plasma Biomarkers in SMNΔ7 Mice following Postnatal SMN Restoration
Source: PLoS One. 2016 Dec 1;11(12):e0167077. doi: 10.1371/journal.pone.0167077 (PMC5132001; doi:10.1371/journal.pone.0167077)
Supplement: S4 Table — P12: ASO-SMA n = 12, scramble SMA n = 13, ASO-Het n = 10, scramble Het n = 5; P30/P90: ASO-SMA n = 12, ASO-Het n = 7, scramble Het n = 8. Shaded boxes represent significant values at p<0.05. (DOCX) [file pone.0167077.s005.docx]

**S5 Table: Correlation of whole blood SMN and plasma analyte levels**

| **Timing** | **P12** | | **P30** | | **P90** | |
| --- | --- | --- | --- | --- | --- | --- |
|  | **r** | **p-value** | **r** | **p-value** | **r** | **p-value** |
| Osteopontin | **-0.454** | **0.004** | **-0.936** | **0.001** | **0.833** | **0.001** |
| DPPIV | **-0.547** | **<0.001** | 0.164 | 0.611 | 0.187 | 0.605 |
| Tetranectin | **0.703** | **<0.001** | 0.056 | 0.864 | 0.096 | 0.791 |
| Fetuin A | 0.161 | 0.334 | 0.261 | 0.412 | 0.492 | 0.148 |
| Vitronectin | **0.526** | **<0.001** | **-0.723** | **0.043** | **0.812** | **0.002** |
