# Supplementary material for: Normalization of Patient-Identified Plasma Biomarkers in SMNΔ7 Mice following Postnatal SMN Restoration
Source: PLoS One. 2016 Dec 1;11(12):e0167077. doi: 10.1371/journal.pone.0167077 (PMC5132001; doi:10.1371/journal.pone.0167077)
Supplement: S5 Table — Biomarker concentration is normalized to total soluble protein (pg/ml). Concentration of SMN protein in tissues is normalized to total soluble protein (pg/mg). Concentration of SMN in whole blood is pg SMN per ml whole blood. CMAP, compound muscle action potential, MUNE, motor unit number estimation, SMUP, single motor unit potential. (DOCX) [file pone.0167077.s006.docx]

# Table S6. Raw data tables for plasma biomarkers, SMN Levels and Electrophysiology at P12, P30, and P90

| **Raw data P12 biomarker levels** | | | |
| --- | --- | --- | --- |
| **P12 AXL** | | | |
| ASO-SMA | SMA | ASO-Het | Het |
| 9725 | 9401 | 12259 | 10951 |
| 7920 | 8545 | 10164 | 8113 |
| 10765 | 9603 | 11989 | 9801 |
| 7246 | 9994 | 7366 | 9830 |
| 10943 | 9757 | 8268 | 10704 |
| 10826 | 12193 | 8412 |  |
| 11603 | 8248 | 9266 |  |
| 10219 | 5433 | 9467 |  |
| 8644 | 9179 | 11071 |  |
| 10161 | 8925 | 9633 |  |
| 8854 | 9620 |  |  |
| 7855 | 9652 |  |  |
|  | 8663 |  |  |
| **P12 Cadherin** | | | |
| ASO-SMA | SMA | ASO-Het | Het |
| 63067 | 42230 |  |  |
| 63059 | 22785 |  |  |
| 12190 | 46545 |  |  |
| 71936 | 34243 |  |  |
| 112087 | 192499 |  |  |
| 13025 | 56039 |  |  |
|  | 50370 |  |  |
|  | 38284 |  |  |
|  | 25107 |  |  |
| **P12 CHI3L1** | | | |
| ASO-SMA | SMA | ASO-Het | Het |
| 88300 | 44005 | 46420 | 36150 |
| 69248 | 35164 | 40266 | 25428 |
| 101390 | 45198 | 60593 | 55416 |
| 43484 | 35266 | 37614 | 58007 |
| 57735 | 40108 | 49682 | 44999 |
| 53190 | 76747 | 43906 |  |
| 56108 | 50879 | 42367 |  |
| 41336 | 29785 | 34910 |  |
| 53489 | 28496 | 57218 |  |
| 53618 | 27926 | 64872 |  |
| 37273 | 33035 |  |  |
| 44753 | 49573 |  |  |
|  | 42607 |  |  |
| **P12 COMP** | | | |
| ASO-SMA | SMA | ASO-Het | Het |
| 151424 | 151260 | 249259 | 196216 |
| 92790 | 129472 | 195713 | 165500 |
| 154136 | 144223 | 213852 | 188572 |
| 125639 | 161765 | 164143 | 205236 |
| 132471 | 159720 | 162851 | 220877 |
| 186715 | 162566 | 174227 |  |
| 170107 | 125462 | 181877 |  |
| 190265 | 93419 | 182028 |  |
| 146594 | 132898 | 182925 |  |
| 157914 | 139779 | 196776 |  |
| 169294 | 142369 |  |  |
| 151001 | 147607 |  |  |
|  | 151522 |  |  |
| **P12 DPPIV** | | | |
| ASO-SMA | SMA | ASO-Het | Het |
| 148949 | 226206 | 173601 | 152964 |
| 64665.8 | 233596 | 142494 | 148665 |
| 162038 | 246547 | 145767 | 149671 |
| 108298 | 253026 | 118370 | 181208 |
| 123519 | 235031 | 140094 | 148597 |
| 143770 | 166271 | 126108 |  |
| 234159 | 221346 | 132363 |  |
| 194283 | 161824 | 131485 |  |
| 164042 | 283724 | 139010 |  |
| 158920 | 291903 | 145335 |  |
| 149583 | 296875 |  |  |
| 122304 | 376085 |  |  |
|  | 249133 |  |  |
| **P12 Fetuin A** | | | |
| ASO-SMA | SMA | ASO-Het | Het |
| 94851800 | 56145020 | 95365130 | 95919080 |
| 44199260 | 58851200 | 95688440 | 81531030 |
| 87490090 | 70316760 | 85937320 | 90348250 |
| 102000000 | 58995820 | 82058000 | 86801940 |
| 117000000 | 58056430 | 83046960 | 93285340 |
| 113000000 | 46963540 | 94113440 |  |
| 98866780 | 80907010 | 79364610 |  |
| 137000000 | 76950470 | 92000480 |  |
| 121000000 | 70442100 | 98406220 |  |
| 132000000 | 63004740 | 99338200 |  |
| 126000000 | 50536340 |  |  |
| 131000000 | 56796840 |  |  |
|  | 58090500 |  |  |
| **P12 IGF-1** | | | |
| ASO-SMA | SMA | ASO-Het | Het |
| 17334 | 6812 | 65048 | 96274 |
| 7490 |  | 77911 | 63667 |
| 7490 |  | 43707 | 100600 |
| 33132 |  | 94422 | 93604 |
| 21948 |  | 88984 | 74280 |
| 37078 |  | 91283 |  |
| 40950 |  | 42943 |  |
| 51301 |  | 69220 |  |
| 11327 |  | 87297 |  |
| 26530 |  | 131653 |  |
| 22421 |  |  |  |
| **P12 Osteopontin** | | | |
| ASO-SMA | SMA | ASO-Het | Het |
| 236314 | 324817 | 327506 | 188376 |
| 172435 | 408971 | 268343 | 132116 |
| 257287 | 365411 | 273010 | 161154 |
| 110976 | 275962 | 133205 | 189743 |
| 151335 | 374967 | 132351 | 195039 |
| 166246 | 534134 | 161023 |  |
| 565960 | 425819 | 169111 |  |
| 154209 | 306773 | 177269 |  |
| 117058 | 363985 | 193127 |  |
| 111555 | 348424 | 218004 |  |
| 147930 | 323718 |  |  |
| 165091 | 438159 |  |  |
|  | 345659 |  |  |
| **P12 Tetranectin** | | | |
| ASO-SMA | SMA | ASO-Het | Het |
| 12269 | 12326 | 11402 | 10755 |
| 13086 | 11879 | 11098 | 9546 |
| 12296 | 14438 | 10518 | 10117 |
| 9809 | 16386 | 10362 | 12738 |
| 11252 | 13774 | 11035 | 12960 |
| 10357 | 15891 | 10494 |  |
| 13802 | 13886 | 9749 |  |
| 11690 | 12135 | 10931 |  |
| 12385 | 13925 | 12877 |  |
| 12865 | 12332 | 10696 |  |
| 12474 | 15241 |  |  |
| 11617 | 14663 |  |  |
|  | 12371 |  |  |
| **P12 Vitronectin** | | | |
| ASO-SMA | SMA | ASO-Het | Het |
| 286344 | 222322 | 343334 | 380580 |
| 146089 | 159759 | 449037 | 339270 |
| 336096 | 207947 | 357882 | 415208 |
| 329224 | 229524 | 336848 | 346133 |
| 302576 | 227062 | 336927 | 327775 |
| 364251 | 137463 | 302301 |  |
| 251587 | 266622 | 297507 |  |
| 304031 | 224515 | 317790 |  |
| 347365 | 202602 | 336624 |  |
| 355697 | 229877 | 313641 |  |
| 317665 | 241572 |  |  |
| 471620 | 199069 |  |  |
|  | 211481 |  |  |
|  |  |  |  |

| **Raw data P12 SMN levels in various tissues** | | | |
| --- | --- | --- | --- |
| **P12 SMN Brain** | | | |
| ASO-SMA | SMA | ASO-Het | Het |
| 19986 | 5242 | 40837 | 30273 |
| 21117 | 4075 | 45670 | 31776 |
| 20205 | 4187 | 43137 | 29541 |
| 20973 | 6363 | 46244 | 23895 |
| 20123 | 5301 | 36262 | 29018 |
| 20704 | 4009 | 42115 |  |
| 7739 | 3053 | 46130 |  |
| 12203 | 3114 | 33241 |  |
| 14552 | 3594 | 49691 |  |
| 10238 | 3919 | 39607 |  |
| 12694 | 4519 |  |  |
| 12014 | 2916 |  |  |
|  | 4522 |  |  |
| **P12 SMN Liver** | | | |
| ASO-SMA | SMA | ASO-Het | Het |
| 18469 | 2143 | 29299 | 21151 |
| 16587 | 1018 | 33717 | 21259 |
| 12342 | 1518 | 37280 | 21652 |
| 13121 | 1184 | 36649 | 20502 |
| 10397 | 1615 | 36974 | 17117 |
| 12463 | 1098 | 36944 |  |
| 1403 | 995 | 37584 |  |
| 3120 | 1025 | 35394 |  |
| 3064 | 1518 | 36548 |  |
| 2176 | 1685 | 23027 |  |
| 3623 | 765 |  |  |
| 2679 | 782 |  |  |
|  | 1125 |  |  |
| **P12 Quadriceps** | | | |
| ASO-SMA | SMA | ASO-Het | Het |
| 9171 | 1496 | 52401 | 44287 |
| 9693 | 653 | 49351 | 23506 |
| 6201 | 2176 | 28662 | 29268 |
| 13593 | 2362 | 35287 | 29353 |
| 12896 | 2640 | 26890 | 49443 |
| 17590 | 1011 | 18912 |  |
| 1291 | 685 | 36248 |  |
| 7701 | 870 | 43970 |  |
| 10804 | 940 | 39540 |  |
| 5907 | 972 | 48619 |  |
| 13536 | 772 |  |  |
| 3410 | 274 |  |  |
|  | 870 |  |  |
| **P12 SMN Spinal Cord** | | | |
| ASO-SMA | SMA | ASO-Het | Het |
| 16891 | 6592 | 35333 | 24392 |
| 22038 | 4934 | 36698 | 23142 |
| 22648 | 7279 | 37884 | 25928 |
| 21177 | 8284 | 41552 | 26788 |
| 18787 | 5676 | 34798 | 27294 |
| 23114 | 5030 | 33743 |  |
| 8440 | 3429 | 42993 |  |
| 12400 | 4449 | 36022 |  |
| 14311 | 4856 | 68907 |  |
| 8468 | 6311 | 32467 |  |
| 13441 | 6120 |  |  |
| 13461 | 5060 |  |  |
|  | 4372 |  |  |

| **Raw Data P12 Whole Blood SMN Levels** | | | |
| --- | --- | --- | --- |
| ASO-SMA | SMA | ASO-Het | Het |
| 106478 | 46275 | 618950 | 517103 |
| 120069 | 34800 | 681853 | 567762 |
| 122967 | 30059 | 806719 | 735404 |
| 111283 | 52854 | 712536 | 721559 |
| 140837 | 55408 | 632551 |  |
| 47721 | 12428 | 649761 |  |
| 158424 | 42094 | 874464 |  |
| 117149 | 42860 | 789637 |  |
| 80323 | 32881 | 811040 |  |
| 130063 | 29558 | 663703 |  |
| 66385 | 24520 |  |  |
|  | 36506 |  |  |
|  | 33486 |  |  |

| **Raw data P30 biomarkers** | | |
| --- | --- | --- |
| **P30 AXL** | | |
| ASO-SMA | ASO-Het | Het |
| 3560 | 3289 | 3270 |
| 3699 | 4031 | 3430 |
| 3291 | 3396 | 2931 |
| 2924 | 3118 | 3381 |
| 3968 | 3266 | 3040 |
| 3303 | 2985 | 3233 |
| 3377 | 2839 | 2714 |
| 3131 |  | 3070 |
| 2030 |  |  |
| 2423 |  |  |
| 2548 |  |  |
| 3406 |  |  |
| **P30 Cadherin** | | |
| All samples below detection limit | | |
| **P30 CHI3L1** | | |
| ASO-SMA | ASO-Het | Het |
| 22368 | 10792 | 14983 |
| 28367 | 18562 | 12225 |
| 25483 | 20078 | 14873 |
| 16511 | 13893 | 12390 |
| 25119 | 9925 | 14302 |
| 36108 | 12283 | 29140 |
| 25512 | 11723 | 27642 |
| 29957 |  | 12019 |
| 21643 |  |  |
| 24852 |  |  |
| 32101 |  |  |
| 23830 |  |  |
| **P30 COMP** | | |
| ASO-SMA | ASO-Het | Het |
| 126418 | 115592 | 107614 |
| 116677 | 136857 | 119778 |
| 110013 | 107999 | 105613 |
| 104688 | 92890 | 109020 |
| 75548 | 91886 | 91913 |
| 98435 | 97514 | 87439 |
| 92304 | 105884 | 110310 |
| 97386 | 104517 |  |
| 79463 |  |  |
| 87157 |  |  |
| 100959 |  |  |
| 86949 |  |  |
| **P30 DPPIV** | | |
| ASO-SMA | ASO-Het | Het |
| 277330 | 228009 | 201878 |
| 262335 | 254223 | 186906 |
| 293377 | 283371 | 231201 |
| 176483 | 215773 | 254321 |
| 246464 | 195418 | 224757 |
| 219077 | 168136 | 237235 |
| 236713 | 219219 | 251026 |
| 237776 |  | 234588 |
| 250468 |  |  |
| 316405 |  |  |
| 258005 |  |  |
| 270588 |  |  |
| **P30 Fetuin A** | | |
| ASO-SMA | ASO-Het | Het |
| 24662490 | 24321110 | 25507490 |
| 23719380 | 19282240 | 19907320 |
| 24327340 | 24101160 | 21922620 |
| 15161480 | 16626030 | 12807900 |
| 16403520 | 19276690 | 12736580 |
| 26782300 | 14199420 | 16131000 |
| 13205950 | 18415320 | 18604990 |
| 31115990 |  | 19837530 |
| 21619930 |  |  |
| 19980380 |  |  |
| 15966230 |  |  |
| 30856150 |  |  |
| **P30 IGF-1** | | |
| ASO-SMA | ASO-Het | Het |
| 102392 | 137126 | 147233 |
| 84510 | 146695 | 117893 |
| 44154 | 137144 | 110772 |
| 42897 | 126496 | 102897 |
| 59208 | 142887 | 194754 |
| 48427 | 103825 | 101981 |
| 91914 | 140393 | 63149 |
| 53743 |  | 110323 |
| 26560 |  |  |
| 23316 |  |  |
| 19186 |  |  |
| 52338 |  |  |
| **P30 Osteopontin** | | |
| ASO-SMA | ASO-Het | Het |
| 103088 | 112546 | 71617 |
| 94759 | 107928 | 109695 |
| 96350 | 90751 | 87088 |
| 92651 | 74079 | 88491 |
| 75641 | 77468 | 102747 |
| 74679 | 74960 | 149607 |
| 69004 | 64651 | 190743 |
| 71149 |  | 72768 |
| 71069 |  |  |
| 73792 |  |  |
| 77729 |  |  |
| 64991 |  |  |
| **P30 Tetranectin** | | |
| ASO-SMA | ASO-Het | Het |
| 12537 | 10040 | 10207 |
| 10816 | 11079 | 10966 |
| 12088 | 10742 | 10057 |
| 10175 | 10453 | 11205 |
| 10778 | 9330 | 10391 |
| 11070 | 9840 | 10430 |
| 10330 | 11245 | 10564 |
| 10229 |  | 10254 |
| 10887 |  |  |
| 11113 |  |  |
| 10735 |  |  |
| 10159 |  |  |
| **P30 Vitronectin** | | |
| ASO-SMA | ASO-Het | Het |
| 408236 | 489982 | 702963 |
| 535983 | 565544 | 500847 |
| 526395 | 555863 | 522119 |
| 874730 | 462569 | 485256 |
| 572885 | 538451 | 501841 |
| 397590 | 508615 | 552627 |
| 405203 | 536106 | 646895 |
| 474709 |  | 415071 |
| 720642 |  |  |
| 555687 |  |  |
| 439621 |  |  |
| 441447 |  |  |

| \| **P30 SMN levels blood** \| \| \| \| --- \| --- \| --- \| \| ASO-SMA \| ASO-Het \| Het \| \| 25875 \| 202693 \| 132836 \| \| 26127 \| 151633 \| 178127 \| \| 21599 \| 138058 \| 152779 \| \| 17101 \| 155974 \| 153165 \| \| 16745 \| 131240 \| 105792 \| \| 25840 \|  \| 97688 \| \| 23453 \|  \| 101165 \| \| 12277 \|  \| 99704 \| \| 15651 \|  \|  \| \| 24661 \|  \|  \| |  |  |
| --- | --- | --- | --- | --- | --- | --- | --- | --- | --- | --- | --- | --- | --- | --- | --- | --- | --- | --- | --- | --- | --- | --- | --- | --- | --- | --- | --- | --- | --- | --- | --- | --- | --- | --- | --- | --- | --- | --- |

| **P90 Biomarkers and SMN Levels** | | |
| --- | --- | --- |
| **P90 AXL** | | |
| ASO-SMA | ASO-Het | Het |
| 1643 | 2239 | 1904 |
| 2037 | 2050 | 2746 |
| 2055 | 2085 | 2362 |
| 1740 | 2190 | 2404 |
| 1489 | 2215 | 1635 |
| 2151 | 2117 | 1717 |
| 4437 | 2114 | 2055 |
| 2282 |  | 1891 |
| 2381 |  |  |
| 2495 |  |  |
| 2710 |  |  |
| 2063 |  |  |
| **P90 CHI3L1** | | |
|  |  |  |
| ASO-SMA | ASO-Het | Het |
| 17100 | 13679 | 14983 |
| 26566 | 16537 | 12225 |
| 15707 | 11993 | 14873 |
| 24235 | 43402 | 12390 |
| 15528 | 14823 | 14302 |
| 28669 | 22030 | 29140 |
| 32672 | 11945 | 27642 |
| 38277 |  | 12019 |
| 27403 |  |  |
| 24585 |  |  |
| 31130 |  |  |
| 53479 |  |  |
| **P90 COMP** | | |
| ASO-SMA | ASO-Het | Het |
| 61644 | 36062 | 23549 |
| 45497 | 37949 | 41714 |
| 46619 | 47790 | 43042 |
| 39248 | 23788 | 55351 |
| 38272 | 52058 | 30198 |
| 47857 | 34165 | 36294 |
| 62432 | 44409 | 27670 |
| 58955 |  | 41231 |
| 57516 |  |  |
| 44682 |  |  |
| 59322 |  |  |
| 48947 |  |  |
| **P90 DPPIV** | | |
| ASO-SMA | ASO-Het | Het |
| 163086 | 276537 | 148209 |
| 201309 | 263423 | 268092 |
| 206063 | 265111 | 258890 |
| 169599 | 71099 | 325949 |
| 101887 | 172911 | 175743 |
| 173034 | 74766 | 224167 |
| 247704 | 179373 | 73700 |
| 258744 |  | 198955 |
| 183111 |  |  |
| 192215 |  |  |
| 180113 |  |  |
| 240348 |  |  |
| **P90 Fetuin A** | | |
| ASO-SMA | ASO-Het | Het |
| 17664060 | 9360601 | 8347840 |
| 14480050 | 8931998 | 13345470 |
| 13848790 | 16404020 | 18852140 |
| 15329650 | 10182510 | 6542173 |
| 6858705 | 8262528 | 7231392 |
| 10924330 | 10536200 | 8503276 |
| 14937940 | 14103890 | 10629670 |
| 10053390 |  | 18524540 |
| 11519010 |  |  |
| 12152600 |  |  |
| 12098040 |  |  |
| 10173070 |  |  |
| **P90 Osteopontin** | | |
| ASO-SMA | ASO-Het | Het |
| 38096 | 67350 | 48681 |
| 41457 | 57218 | 67190 |
| 40870 | 46394 | 79336 |
| 43770 | 77618 | 75426 |
| 55107 | 63314 | 52692 |
| 37139 | 73831 | 53907 |
| 53326 | 35732 | 118228 |
| 45751 |  | 41631 |
| 33471 |  |  |
| 30184 |  |  |
| 28224 |  |  |
| 33754 |  |  |
| **P90 Tetranectin** | | |
| ASO-SMA | ASO-Het | Het |
| 12050 | 7704 | 7786 |
| 9168 | 9302 | 8624 |
| 9055 | 10684 | 8827 |
| 10162 | 6243 | 8163 |
| 8022 | 8265 | 6962 |
| 9429 | 7598 | 8446 |
| 10429 | 8797 | 7909 |
| 9776 |  | 7886 |
| 10614 |  |  |
| 9300 |  |  |
| 10617 |  |  |
| 10240 |  |  |
| **P90 IGF-1** | | |
| ASO-SMA | ASO-Het | Het |
| 245311 | 81996 | 71448 |
| 79481 | 115057 | 88299 |
| 81560 | 76480 | 70907 |
| 90284 | 89304 | 43736 |
| 77159 | 115736 | 128258 |
| 43055 | 106558 | 96760 |
| 78286 | 99068 | 53733 |
| 46097 |  | 68886 |
| 79067 |  |  |
| 64774 |  |  |
| 50566 |  |  |
| 46268 |  |  |
| **P90 Vitronectin** | | |
| ASO-SMA | ASO-Het | Het |
| 550457 | 549373 | 326090 |
| 515701 | 638405 | 750137 |
| 532175 | 499358 | 435373 |
| 561380 | 1052738 | 613546 |
| 566163 | 583005 | 558874 |
| 586159 | 861375 | 514951 |
| 475183 | 500569 | 711798 |
| 489537 |  | 476709 |
| 447181 |  |  |
| 418745 |  |  |
| 407738 |  |  |
| 560342 |  |  |
| **P90 Cadherin** | | |
| All samples below detection limit | | |

| **Raw data P90 SMN levels in various tissues** | | |
| --- | --- | --- |
| **P90 SMN Brain** | | |
| ASO-SMA | ASO-Het | Het |
| 2259 | 8169 | 7635 |
| 2421 | 7873 | 6709 |
| 2458 | 8480 | 7223 |
| 1853 | 8153 | 5858 |
| 2214 | 9161 | 7636 |
| 1401 | 8175 | 5418 |
| 1829 | 9226 | 5330 |
| 1420 |  | 6230 |
| 2570 |  |  |
| 2396 |  |  |
| 4208 |  |  |
| 2492 |  |  |
| **P90 SMN Liver** | | |
| ASO-SMA | ASO-Het | Het |
| 1943 | 10824 | 10328 |
| 3131 | 9995 | 7763 |
| 3093 | 9472 | 8640 |
| 4551 | 10364 | 7317 |
| 3553 | 8959 | 9669 |
| 2187 | 10400 | 8525 |
| 1749 | 9716 | 9230 |
| 2301 |  | 6531 |
| 4115 |  |  |
| 2839 |  |  |
| 4647 |  |  |
| 974 |  |  |
| **P90 SMN Quadriceps** | | |
| ASO-SMA | ASO-Het | Het |
| 348 | 997 | 1048 |
| 655 | 880 | 836 |
| 397 | 834 | 845 |
| 264 | 1434 | 819 |
| 505 | 395 | 981 |
| 638 | 1187 | 637 |
| 741 | 674 | 631 |
| 184 |  | 470 |
| 487 |  |  |
| 460 |  |  |
| 789 |  |  |
| 281 |  |  |
| **P90 SMN Spinal Cord** | | |
| ASO-SMA | ASO-Het | Het |
| 1260 | 3909 | 3200 |
| 2625 | 2533 | 3364 |
| 1477 | 2937 | 2454 |
| 1741 | 3653 | 3495 |
| 1635 | 2967 | 4742 |
| 3596 | 4175 | 3190 |
| 2014 | 3038 | 4155 |
| 2262 |  | 4282 |
| 3269 |  |  |
| 3296 |  |  |
| 4117 |  |  |
| 2019 |  |  |
|  |  |  |

| **Raw data P90 SMN levels blood** | | |
| --- | --- | --- |
| ASO-SMA | ASO-Het | Het |
| 13436 | 89531 | 45971 |
| 18720 | 115580 | 100177 |
| 11270 | 517871 | 107427 |
| 11223 | 113547 | 81652 |
| 22452 | 371784 | 66323 |
| 33221 | 78031 | 60079 |
| 13248 |  | 66313 |
| 29881 |  | 50450 |
| 21672 |  |  |
| 18956 |  |  |
| 22098 |  |  |

| **Raw data electrophysiology data** | | | | |
| --- | --- | --- | --- | --- |
| **P12 Electrophysiology** | | | | |
| Mouse number | Group | P12 Sciatic CMAP mV | P12 MUNE | P12 SMUP µV |
| 8 | ASO-SMA | 19.6 | 244 | 136.2 |
| 2 | ASO-SMA | 17.9 | 227 | 142.8 |
| 3 | ASO-SMA | 19.1 | 219 | 169.7 |
| 6 | ASO-SMA | 14.3 | 40 | 769.5 |
| 18 | ASO-SMA | 15.4 | 282 | 106.2 |
| 13 | ASO-Het | 14.2 | 270 | 106.8 |
| 14 | ASO-Het | 20.9 | 489 | 83 |
| 15 | ASO-Het | 25.8 | 328 | 164.2 |
| 16 | ASO-Het | 20.5 | 213 | 185.8 |
| 21 | ASO-Het | 22.1 | 106 | 424 |
| 22 | ASO-Het | 19.2 | 94 | 415.7 |
| 23 | ASO-Het | 26.4 | 291 | 163.3 |
| 24 | ASO-Het | 25 | 145 | 328.2 |
| **P30 Electrophysiology** | | | | |
|  | Group | P30 Sciatic CMAP mV | P30 MUNE | P30 SMUP µV |
| 8 | ASO-SMA | 22.9 | 94 | 824.7 |
| 2 | ASO-SMA | 37.5 | 453 | 152.7 |
| 3 | ASO-SMA | 29.3 | 158 | 313.5 |
| 6 | ASO-SMA | 37.2 | 181 | 312 |
| 18 | ASO-SMA | 24.5 | 102 | 430.8 |
| 13 | ASO-Het | 35.9 | 272 | 256.8 |
| 14 | ASO-Het | 36.1 | 291 | 235.3 |
| 15 | ASO-Het | 36.7 | 210 | 353.2 |
| 16 | ASO-Het | 39 | 333 | 235.2 |
| 21 | ASO-Het | 33.1 | 482 | 150.2 |
| 22 | ASO-Het | 37.1 | 191 | 349.7 |
| 23 | ASO-Het | 42.5 | 381 | 194.3 |
| 24 | ASO-Het | 27.5 | 416 | 141.2 |
| **P90 Electrophysiology** | | | | |
|  | Group | P90 Sciatic CMAP mV | P90 MUNE | P90 SMUP µV |
| 8 | ASO-SMA | 56.1 | 211 | 374.7 |
| 2 | ASO-SMA | 47.7 | 34 | 2051.8 |
| 3 | ASO-SMA | 46.8 | 183 | 381.7 |
| 6 | ASO-SMA | 41.4 | 240 | 300.2 |
| 18 | ASO-SMA | 41.2 | 284 | 219.7 |
| 14 | ASO-Het | 49 | 225 | 384.7 |
| 15 | ASO-Het | 54.2 | 272 | 352.3 |
| 16 | ASO-Het | 51.3 | 291 | 314.3 |
| 21 | ASO-Het | 52.1 | 205 | 352.8 |
| 24 | ASO-Het | 49.3 | 252 | 233.2 |
